# Views on life and death of physicians, nurses, cancer patients and general population in Japan

**DOI:** 10.1371/journal.pone.0176648

**Published:** 2017-05-03

**Authors:** Noriyasu Sekiya, Yujiro Kuroda, Kasumi Nakajima, Yumi Iwamitsu, Yoshiaki Kanai, Mitsunori Miyashita, Midori Kotani, Yutaka Kitazawa, Hideomi Yamashita, Keiichi Nakagawa

**Affiliations:** 1 Department of Radiology, University of Tokyo Hospital, Tokyo, Japan; 2 Department of Public Health Fukushima Medical University School of Medicine, Fukushima City, Japan; 3 Faculty of Health Sciences, University of Tokyo Health Sciences, Tokyo, Japan; 4 Department of Medical Psychology, Graduate School of Medical Sciences, Kitasato University, Tokyo, Japan; 5 Department of Palliative Medicine, University of Tokyo Hospital, Tokyo, Japan; 6 Division of Palliative Nursing, Health Sciences, Tohoku University Graduation School of Medicine, Sendai, Miyagi, Japan; 7 Dai-ichi Life Research Institute Inc., Tokyo, Japan; 8 Toyo Eiwa University, Tokyo, Japan; Osaka University Graduate School of Medicine, JAPAN

## Abstract

This study aimed to investigate views on life and death among physicians, nurses, cancer patients, and the general population in Japan and examine factors affecting these views. We targeted 3,140 physicians, 470 nurses, 450 cancer patients, and 3,000 individuals from the general population. We used the Death Attitudes Inventory (DAI) to measure attitudes toward life and death. The collection rates were 35% (1,093/3,140), 78% (366/470), 69% (310/450), and 39% (1,180/3,000) for physicians, nurses, patients, and the general population, respectively. We found that age, sex, social role (i.e., physician, nurse, cancer patient, and general population) were significantly correlated with DAI subscales. Compared with general population, attitudes toward death of physicians, nurses and cancer patients differed significantly even after adjusted their age and sex. Our study is the first to analyze differences in views on life and death among physicians, nurses, cancer patients, and the general population in Japan.

## Introduction

End-of-life decision making is affected by cultural and religious factors [1–4]. However, cultural and religious beliefs vary; therefore, it is unclear what specific beliefs affect end-of-life decisions. Terror management theory (TMT), proposed by Greenberg, Solomon, and Pyszczynski, posits that human awareness of the inevitability of death exerts a profound influence on human thought, emotion, motivation, and behavior and people manage the potential for anxiety that results from this awareness by maintaining: (1) faith in the absolute validity of their cultural worldviews and (2) self-esteem by living up to the standards of values that are part of their worldviews [5]. Experimental evidence from studies conducted in Western countries and Japan supports TMT [6,7]. Based on TMT, we hypothesized that views on life and death promoted by one’s culture and religion fundamentally affect health-related decision-making. However, few studies have investigated views on life and death in the Japanese population from this perspective.

The Death Attitude Inventory (DAI) developed by Hirai measures Japanese views on life and death [8]. DAI is a measurement for death attitude which is expected to clarify the structure of death attitude and death-related issues of Japanese. One of our coauthors published a study that used the DAI to investigate views on life and death in Japanese cancer patients [9]. Following this study, we expanded our research to include physicians, nurses, and the general population in addition to cancer patients.

## Materials and methods

### Participants

A total of 3,140 physicians, 470 nurses, 450 cancer patients, and 3,000 individuals from the general population participated in our study. The physicians comprised 2,985 general practitioners in five areas of Japan (Edogawa ward, Omori ward, Nakano ward, Himeji city, and Matsuyama city) and 155 specialists at the University of Tokyo Hospital. All nurses were staff at the University of Tokyo Hospital. Cancer patients were those receiving outpatient treatment in the radiology department at the University of Tokyo Hospital. Some of them underwent not only radiotherapy but chemotherapy and/or surgery up to their clinical conditions. As for the general population, we chose 3,000 potential participants aged 20–70 years in the Tokyo metropolitan area using a stratified two-stage random sampling method. We entrusted the survey of general population to Shin Joho Center, which is a research organization authorized by the Japanese government in 1972. The study protocol was approved by the ethical review board of the Faculty of Medicine at the University of Tokyo.

### Survey methodology

We conducted a questionnaire survey. The questionnaire included the DAI, items concerning the participant’s demographics, and end-of-life care preferences. As for end-of-life care preferences, we asked patients where they would like to spend their final days and whether or not they would like to be told how long they have left to live. These results were reported previously [9]. We also questioned physicians regarding their religious beliefs. We distributed questionnaires among physicians, nurses, and cancer patients. The completed questionnaires from physicians and nurses were returned via post while those from cancer patients were returned via post or turned in to their doctors. The general population was contacted via E-mail. The investigation periods were between January 2007 and September 2014, between January and December of 2007, between January and August of 2008, and between September 2008 and September 2009, for physicians, nurses, cancer patients, and the general population, respectively. One year after the survey, we conducted a follow-up survey to determine the survival status of the participants.

### Measures

The DAI has seven subscales: views on an afterlife; death anxiety/fear; death as a release; avoidance of death; sense of purpose in life; interest in death; view on a predestined lifespan. The views on a predestined life span subscale has 3 items; all other subscales have 4 items. Each item is scored on a 7-point Likert scale.

The subscales were interpreted as follows: (1) a higher score on “view on an afterlife” indicated a stronger belief in the existence of life after death; (2) a higher score on “death anxiety/fear” indicated a greater fear of death or a stronger propensity for thoughts of death to trigger anxiety; (3) a higher score on “death as a release” indicated a stronger sense of death as a release from pain and suffering; (4) a higher score on “avoidance of death” indicated a stronger tendency to avoid thinking about death; (5) a higher score on “sense of purpose” indicated that the patient had found a clear sense of purpose or a goal in their life; (6) a higher score on “interest in death” indicated a more frequent tendency to think about one’s own death or the death of members of one’s social circle; and (7) a higher score on “view on a predestined lifespan” indicated a stronger belief that our lifespan is determined from the outset or that our life and death are determined by an invisible force (e.g., fate or a divine being).

The test-retest reliability, internal consistency, factor validity, concurrent validity, and construct validity of this measure were confirmed by the original author in a small sample (n = 79) [8].

### Statistical analysis

All analyses were conducted using R (v. 3.3.0). To evaluate the psychometric properties of the DAI, we calculated the mean; standard deviation (SD); skewness; kurtosis; floor and ceiling effects; and the number of missing items. We calculated kurtosis as (m^4^ / s^4^) - 3, where m for the sample moments and s for the sample standard deviation[10]. We regarded a kurtosis of less than 7 and a skewness of less than 2 as sufficient normality.

We then conducted confirmatory factor analysis (CFA) on the DAI. First, we compared two CFA models, with and without the assumption that the latent variables were correlated. Then, we fitted a second-order CFA model that reflected TMT to investigate whether the DAI could be conceptually connected with TMT. The model fit was assessed by several goodness-of-fit indices: the chi-square goodness-of-fit statistic, the comparative fit index (CFI), the Tucker–Lewis index (TLI), the root mean square error of approximation (RMSEA), and the standardized root mean square residual (SRMR). CFI and TLI values of 0.90 or greater were interpreted as an acceptable fit and a value of 0.08 or less was regarded as a good fit for the RMSEA and SRMR[11,12].

A multi-group CFA was performed to test the measurement invariance across physicians, nurses, patients, and the general population. We adapted the ⊿CFI (<0.01) as the criterion to identify a significant decrease in the fit among a series of progressive restricted models. Based on the measurement invariance of the DAI across the abovementioned groups, we developed a structural equation model (SEM) to investigate the effects of age, sex, and group on the DAI subscales.

## Results

### Participant demographics

The collection rates were 35% (1,093/3,140), 78% (366/470), 69% (310/450), and 39% (1,180/3,000) for physicians, nurses, patients, and the general population, respectively. Age, sex, and other demographic characteristics of the participants are shown in Table 1. There were more male than female physicians (82.3% vs. 17.1%); most nurses were young females. Most of our patients were cancer survivors, and at least 79.0% of them were alive at the 1-year follow-up.

**Table 1:** Participant demographics.

		Physicians		Nurses		Patients		General population	
		n	%	n	%	n	%	n	%
Age, n, %									
	20–29	14	1.3%	237	64.8%	6	1.9%	114	9.7%
	30–39	69	6.3%	73	19.9%	17	5.5%	229	19.4%
	40–49	221	20.2%	26	7.1%	34	11.0%	202	17.1%
	50–59	348	31.8%	22	6.0%	61	19.7%	208	17.6%
	60–69	237	21.7%	1	0.3%	90	29.0%	256	21.7%
	70–79	179	16.4%			77	24.8%	153	13.0%
	80–89	17	1.6%			23	7.4%		
	> 90					2	0.6%		
	NA	8	0.7%	7	1.9%			18	1.5%
Sex, n, %									
	Male	900	82.3%	15	4.1%	182	58.7%	486	41.2%
	Female	187	17.1%	345	94.3%	128	41.3%	674	57.1%
	NA	6	0.5%	6	1.6%			20	1.7%
Professional Career, n, %									
	< 1year	2	0.2%	53	14.5%				
	1–3 years	9	0.8%	116	31.7%				
	3–5 years	8	0.7%	38	10.4%				
	5–10 years	30	2.7%	66	18.0%				
	10–15 years	58	5.3%	31	8.5%				
	15–19 years	100	9.1%	20	5.5%				
	20–29 years	322	29.5%	23	6.3%				
	> 30years	557	51.0%	13	3.6%				
	NA	7	0.6%	6	1.6%				
KPS, mean(SD)						91	10		
Treatment Status, n, %									
	before treatment					11	3.5%		
	radical treatment					34	11.0%		
	palliative treatment					19	6.1%		
	follow up					230	74.2%		
	Other					16	5.2%		
Survival Status 1 year later, n, %									
	Alive					245	79.0%		
	Dead					47	15.2%		
	Unknown					18	5.8%		

We summarized the responses for each DAI item and subscale as means and SD in Table 2.

**Table 2:** DAI item responses of physicians, nurses, patients, and the general population.

	Physicians			Nurses			Patients			General population		Total	
	Mean	SD	NBS	Mean	SD	NBS	Mean	SD	NBS	Mean	SD	Mean	SD
Afterlife view	11.2	6.9	43.4	17.2	5.1	52.8	13.8	6.8	47.6	15.4	6.3	13.9	6.8
I believe there is an afterlife	2.9	2.1	43.9	4.4	1.5	52.4	3.7	2.0	48.2	4.0	1.8	3.6	2.0
I believe there are spirits or curses in this world	2.8	1.9	44.3	4.2	1.5	52.1	3.4	1.8	48.0	3.8	1.8	3.4	1.9
I believe your soul remains after you die	3.0	2.0	44.6	4.3	1.4	52.2	3.6	1.9	48.1	3.9	1.8	3.6	1.9
I believe that people are reborn after death	2.5	1.8	44.1	4.3	1.5	53.2	3.2	1.9	47.6	3.7	1.9	3.3	1.9
Death anxiety/fear	15.2	6.9	46.3	17.0	6.2	49.2	16.7	7.2	48.7	17.5	6.3	16.5	6.7
Dying is frightening	4.2	1.9	46.5	4.8	1.7	49.7	4.5	1.9	47.9	4.8	1.7	4.6	1.8
I become anxious when I think about dying	4.0	1.9	46.9	4.5	1.8	49.5	4.4	1.9	49.1	4.6	1.7	4.3	1.9
I think that death is frightening	3.6	1.9	46.5	4.1	1.6	49.4	4.0	2.0	49.2	4.2	1.7	3.9	1.8
I am extremely frightened of death	3.4	1.9	46.7	3.7	1.7	48.5	3.8	1.9	49.3	4.0	1.8	3.7	1.8
Death as a release	12.7	6.7	49.6	13.1	5.5	50.2	13.6	6.7	51.0	13.0	6.3	13.0	6.4
I believe that death is a release from suffering in this world	3.3	1.9	49.8	3.4	1.5	50.3	3.4	1.8	50.8	3.3	1.7	3.3	1.8
I believe that death is a release from the burdens of this life	3.1	1.8	49.9	3.1	1.5	50.0	3.2	1.8	50.2	3.1	1.7	3.1	1.8
Death is a release from pain and suffering	3.4	1.9	49.8	3.4	1.5	49.6	3.7	1.9	51.3	3.5	1.8	3.5	1.8
Death brings a release for the soul	2.9	1.8	48.7	3.3	1.5	50.6	3.3	1.8	51.0	3.2	1.7	3.1	1.7
Death avoidance	10.6	5.4	45.7	10.7	5.1	45.9	13.6	6.5	51.0	13.1	5.7	11.9	5.7
I avoid thinking about death	3.0	1.7	47.1	3.0	1.4	46.8	3.5	1.8	50.4	3.5	1.6	3.3	1.7
I would prefer to avoid thinking about death at all costs	2.3	1.5	45.0	2.5	1.4	46.2	3.2	1.9	50.5	3.2	1.7	2.8	1.6
Whenever thoughts about death come up, I try to throw them off	2.6	1.6	46.5	2.6	1.4	46.3	3.4	1.8	51.6	3.2	1.7	2.9	1.7
I try not to think about death because it is scary	2.7	1.6	46.3	2.7	1.5	46.6	3.4	1.8	50.8	3.3	1.7	3.0	1.7
Sense of purpose in life	17.0	5.3	51.8	15.8	4.4	49.5	16.5	5.3	50.8	16.1	5.0	16.4	5.1
I have found a clear purpose and goal in life	4.5	1.7	53.1	3.8	1.3	48.6	4.1	1.6	50.5	4.0	1.5	4.2	1.6
I have the necessary capabilities to find meaning, purpose, and a goal	4.2	1.6	53.2	3.7	1.3	50.1	3.8	1.6	50.6	3.7	1.5	3.9	1.5
When I think about life, I have a clear reason for what I am doing with it	4.2	1.7	51.2	3.9	1.4	48.9	4.3	1.6	51.7	4.0	1.6	4.1	1.6
The future is bright	4.1	1.4	48.5	4.4	1.2	50.9	4.3	1.6	49.7	4.3	1.4	4.2	1.4
Interest in death	14.0	5.7	50.3	16.0	5.1	54.1	14.2	5.8	50.8	13.8	5.4	14.2	5.6
I often wonder what death is all about	3.5	1.8	49.6	4.2	1.6	54.1	3.5	1.8	50.0	3.5	1.7	3.6	1.8
I often think about my own death	3.8	1.8	51.4	4.0	1.6	52.6	3.8	1.8	51.7	3.5	1.7	3.7	1.8
I often think about people close to me dying	3.9	1.8	50.2	4.4	1.5	53.3	4.0	1.7	50.9	3.8	1.7	3.9	1.7
I often think about my friends and family dying	2.9	1.6	49.6	3.5	1.6	53.3	3.0	1.7	50.3	2.9	1.6	3.0	1.6
View on a predestined lifespan	10.4	5.5	48.2	11.4	4.5	50.1	11.9	5.3	51.0	11.4	5.1	11.1	5.2
I believe that lifespan is determined in advance	3.7	2.0	48.6	4.0	1.6	50.3	4.1	2.0	50.9	3.9	1.9	3.9	1.9
I believe that lifespan is determined from the outset	3.4	2.0	48.4	3.8	1.7	50.4	3.9	1.9	51.0	3.7	1.8	3.7	1.9
I believe that life and death are determined by an invisible force (fate)	3.3	2.0	48.0	3.7	1.6	49.8	3.9	1.9	51.1	3.7	1.9	3.6	1.9

SD: standard deviation. NBS: norm-based score. NBS = {(raw score − mean of the general population)/SD of the general population} × 10 + 50.

### Confirmatory factory analysis

We examined the seven first-order factors of the DAI in the total sample (Table 3).

**Table 3:** Primary goodness-of-fit comparative indices for the DAI model and the structural invariance of the multi-group model.

Model	Type	χ	p	df	CFI	TLI	RMSEA	SRMR
The total sample	Uncorrelated	4478.12	***	324	0.92	0.91	0.07	0.15
	Correlated	2523.49	***	303	0.96	0.95	0.05	0.05
	Second-order factors	3139.30	***	318	0.94	0.94	0.06	0.08

**: p < 0.001.

df: degree of freedom. CFI: comparative fit index. TLI: Tucker–Lewis index. RMSEA: root mean square error of approximation. SRMR: standardized root mean square residual.

The model that assumed a correlation between the latent variables showed a better fit, indicating that the seven factors of the DAI were correlated. In this model, both the CFI and TLI were greater than 0.90 and both the RMSEA and SRMR were less than 0.08. This supports the original seven-factor structure proposed by Hirai et al. The standardized factor loadings were all significant and ranged from 0.48 (subscale 5 to “The future is bright.”) to 0.96 (subscale 3 to “I believe that death is a release from the burdens of this life.”)(Table 4). These results suggest that the items converged significantly on the subscales. The correlation matrix of the subscales is shown in Table 5.

**Table 4:** Corrected item-scale correlation and factor loadings for items of the DAI.

Subscales	Items	r	factor loadings	
Afterlife view	I believe there is an afterlife	0.86	0.88	***
	I believe there is an afterlife	0.81	0.82	***
	I believe your soul remains after you die	0.88	0.90	***
	I believe that people are reborn after death	0.81	0.83	***
Death anxiety/fear	Dying is frightening	0.86	0.87	***
	I become anxious when I think about dying	0.88	0.89	***
	I think that death is frightening	0.88	0.89	***
	I am extremely frightened of death	0.85	0.86	***
Death as a release	I believe that death is a release from suffering in this world	0.9	0.92	***
	I believe that death is a release from the burdens of this life	0.93	0.96	***
	Death is a release from pain and suffering	0.87	0.86	***
	Death brings a release for the soul	0.76	0.74	***
Death avoidance	I avoid thinking about death	0.75	0.76	***
	I would prefer to avoid thinking about death at all costs	0.83	0.84	***
	Whenever thoughts about death come up, I try to throw them off	0.81	0.84	***
	I try not to think about death because it is scary	0.83	0.86	***
Sense of purpose in life	I have found a clear purpose and goal in life	0.84	0.87	***
	I have the necessary capabilities to find meaning, purpose, and a goal	0.86	0.91	***

***: p < 0.001.

r: item by scale correlations corrected for item overlap and scale reliability

**Table 5:** Intercorrelations of the seven DAI subscales.

		1	2	3	4	5	6	7
1	Afterlife view	-						
2	Death anxiety/fear	0.17	-					
3	Death as a release	0.14	0.00	-				
4	Death avoidance	0.14	0.55	0.18	-			
5	Sense of purpose in life	0.13	-0.04	0.02	-0.01	-		
6	Interest in death	0.22	0.25	0.25	0.02	0.18	-	
7	View on a predestined lifespan	0.34	0.03	0.25	0.14	0.09	0.19	-

To explore the connectedness ability of the DAI with TMT, we developed a second-order factorial model. We divided items into those conceptualizing consciousness and those conceptualizing unconsciousness, similar to that in the TMT. We grouped death anxiety/fear, death avoidance, and interest in death as one factor: conscious constructs related to death were grouped and unconscious constructs were grouped separately. Both the CFI and TLI were greater than 0.09 and the RMSEA was 0.06. Only the SRMR was slightly greater than 0.08. Although the second-order model showed slightly decreased fit in comparison with the first-order model with factorial correlation, its fit was still acceptable.

### Measurement Invariance

We performed a multi-group CFA with progressively restricted specifications on the seven-factor model to examine the measurement invariance across groups (i.e., physicians, nurses, patients, and the general population). A series of models were fitted: configural, weak, strong, and strict invariance. Configural invariance means that the same factor structure underlies all groups. The weak invariance model tests if the factor loadings can be constrained to be equal across groups. The strong invariance model tested if the factor loadings and intercepts could be constrained to be equal across the groups. The strict invariance model tested if the factor loadings, intercepts, and residual variances could be constrained to be equal across groups. Each model was nested in the previous model. We summarized the results in Table 6.

**Table 6:** Structural invariance for the nested sequence of multi-group models.

Model	Type	Chisq diff		CFI	RMSEA	⊿CFI	⊿RMSEA
Group							
	Configural			0.951	0.056	NA	NA
	Weak	145.41	***	0.949	0.055	0.002	0
	Strong	395.77	***	0.943	0.058	0.007	0.002
	Strict	782.7	***	0.929	0.062	0.014	0.005

***: p < 0.001.

Chisq: chi-square. Chisq-diff: chi-square difference. CFI: comparative fit index. RMSEA: root mean square error of approximation.

The chi-square difference tests showed statistically significant reductions in the fit of nested models compared with their previous model. We used ⊿CFI < 0.01 and ⊿RMSEA < 0.015 as cutoff points to judge the equivalence across groups. According to this criterion, strong measurement equivalence across groups was demonstrated. As for strong measurement invariance, the ⊿CFI was greater than 0.01 while the ⊿RMSEA was less than 0.015. Therefore, we could assume the same factor structure and factor loadings across groups.

### Correlation of sex, age, and group with the DAI

We used a semantic equation model to investigate the correlation of age, sex, and group to the seven subscales of the DAI. We used male as the baseline of sex, and the general population as that of the groups. The results are shown in Table 7.

**Table 7:** Regression analysis predicting seven subscales of the DAI from age, sex, and group.

	Afterlife view		Death anxiety/fear		Death as a release		Death avoidance		Sense of purpose in life		Interest in death		Predestined lifespan view	
	β		β		β		β		β		β		β	
Age	-0.19	***	-0.20	***	0.21	***	0.07	**	-0.01		-0.10	***	0.17	***
Sex	0.19	***	-0.01		0.12	***	-0.06	*	0.00		0.07	**	0.21	***
Physician	-0.21	***	-0.14	***	-0.01		-0.26	***	0.15	***	0.07	**	-0.03	*
Nurse	-0.05	*	-0.11	***	0.07	**	-0.10	***	-0.02	*	0.09	**	0.03	**
Patient	-0.02	*	0.01		-0.02	*	0.01		0.02	*	0.05	*	0.00	

***:p < 0.001,

**:p < 0.01,

*:p < 0.05,

^+^:p < 0.1.

Older age was positively correlated with the “death as a release,” “death avoidance,” and “predestined lifespan view” subscales and negatively correlated with the “afterlife view,” “death anxiety/fear,” and “interest in death” subscales. The female sex showed a positive correlation with the “afterlife view,” “death as a release,” “interest in death,” and “predestined lifespan view” subscales and negative correlation to the “death avoidance” subscale. The physician group showed positive correlations with the “sense of purpose in life” and “interest in death” subscales and negative correlations with the “afterlife view,” “death anxiety/fear,” “death avoidance,” and “predestined lifespan view” subscales. The nurses group showed positive correlations with the “death as a release,” “interest in death,” and “predestined lifespan view” subscales and negative correlations with the “afterlife view,” “death anxiety/fear,” “death avoidance,” and “;sense of purpose in life” subscales. Cancer patients showed positive correlations with the “;sense of purpose in life” and “interest in death” subscales and negative correlation with the “afterlife view” and “death as a release” subscales. Physicians and cancer patients demonstrated the same pattern of positive correlations.

### Correlation of sex, age, and faith in physicians

The status of faith among physicians was as follows: have faith (n = 385; 35%), no faith (n = 698; 64%), and not available (n = 10; 1%). The breakdown of religion in those who had faith was Buddhism or Shintoism (n = 272; 68.3%), Christianity (n = 43; 10.9%), no specific religion (n = 55; 13.9%), other (n = 10; 2.5%), and not available (n = 15; 3.8%). We conducted a path analysis for the physician data to explain the subscales of the DAI by faith while controlling for age and sex as residual variables. This time, those who did not have faith were regarded as the baseline. The analysis showed that physicians who had faith showed positive correlations with the “afterlife view” (p < 0.01), “death as a release” (p = 0.05), “sense of purpose in life” (p = 0.01), “interest in death” (p < 0.01), and “predestined lifespan view” (p < 0.01) subscales. There was no statistically significant difference in the “death anxiety/fear” and “death avoidance” subscales. The results are shown in Table 8.

**Table 8:** Regression analysis predicting the seven subscales of the DAI from age, sex, and faith in physicians.

	Afterlife view		Death anxiety/fear		Death as a release		Death avoidance		Sense of purpose in life		Interest in death		Predestined lifespan view	
	β		β		β		β		β		β		β	
Age	-0.22	***	-0.24	***	0.10	**	-0.02		-0.01		-0.14	***	0.07	*
Sex	0.10	**	-0.07	*	0.09	**	-0.10	**	0.05	*	0.09	**	0.17	***
Faith	0.31	***	0.01		0.06	*	-0.01		0.09	*	0.12	***	0.14	***

***:p < 0.001,

**:p < 0.01,

*:p < 0.05,

^+^:p < 0.1.

### Item-metric analysis

Means, SD, skewness, kurtosis, proportion of floor effect, ceiling effect, and missing responses, and Cronbach’s α of each item and subscale of the DAI are summarized in Table 9.

**Table 9:** Mean, standard deviation, skewness, kurtosis, floor, and ceiling for the subscales and items of the DAI.

Subscales	Items	Mean	SD	Skewness	Kurtosis	Floor	Ceiling	NA	Cronbach's α
Afterlife view		13.90	6.84	0.02	-0.92	16.6%	3.0%	4.0%	0.91
	I believe there is an afterlife	3.60	1.96	0.08	-1.05	24.6%	10.3%	2.4%	
	I believe there are spirits or curses in this world	3.42	1.87	0.12	-1.05	25.7%	6.6%	2.3%	
	I believe your soul remains after you die	3.60	1.91	0.05	-1.02	22.7%	8.7%	2.8%	
	I believe that people are reborn after death	3.27	1.93	0.28	-1.01	29.9%	7.7%	2.7%	
Death anxiety/fear		16.51	6.69	-0.21	-0.71	7.4%	5.8%	4.7%	0.93
	Dying is frightening	4.56	1.84	-0.46	-0.62	10.4%	18.0%	2.7%	
	I become anxious when I think about dying	4.32	1.85	-0.33	-0.83	11.5%	13.9%	2.9%	
	I think that death is frightening	3.93	1.83	-0.09	-0.84	15.2%	10.2%	2.8%	
	I am extremely frightened of death	3.69	1.84	0.08	-0.92	17.4%	8.7%	3.7%	
Death as a release		12.96	6.39	0.25	-0.65	15.9%	2.5%	4.0%	0.93
	I believe that death is a release from suffering in this world	3.30	1.77	0.25	-0.79	23.4%	5.5%	2.7%	
	I believe that death is a release from the burdens of this life	3.12	1.75	0.37	-0.76	26.4%	4.7%	2.8%	
	Death is a release from pain and suffering	3.46	1.81	0.15	-0.90	21.4%	6.7%	2.9%	
	Death brings a release for the soul	3.11	1.73	0.30	-0.75	27.5%	4.3%	3.4%	
Death avoidance		11.89	5.74	0.39	-0.40	15.0%	0.9%	4.1%	0.89
	I avoid thinking about death	3.25	1.65	0.22	-0.65	20.9%	3.9%	3.1%	
	I would prefer to avoid thinking about death at all costs	2.77	1.64	0.62	-0.37	31.1%	3.1%	3.1%	
	Whenever thoughts about death come up, I try to throw them off	2.90	1.65	0.52	-0.54	27.6%	3.2%	2.8%	
	I try not to think about death because it is scary	3.00	1.68	0.44	-0.65	26.5%	3.6%	2.7%	
Sense of purpose in life		16.41	5.07	-0.15	0.11	2.3%	2.2%	4.5%	0.85
	I have found a clear purpose and goal in life	4.19	1.56	-0.21	-0.25	7.3%	8.4%	3.2%	
	I have the necessary capabilities to find meaning, purpose, and a goal	3.89	1.53	-0.12	-0.23	9.4%	5.6%	3.4%	
	When I think about life, I have a clear reason for what I am doing with it	4.09	1.61	-0.19	-0.37	9.1%	7.9%	3.1%	
	The future is bright	4.23	1.41	-0.19	0.37	5.9%	7.6%	3.0%	
Interest in death		14.19	5.57	-0.01	-0.52	6.4%	1.0%	4.2%	0.83
	I often wonder what death is all about	3.60	1.75	0.11	-0.84	15.6%	6.4%	3.0%	
	I often think about my own death	3.71	1.75	0.03	-0.89	14.0%	6.5%	2.9%	
	I often think about people close to me dying	3.91	1.70	-0.16	-0.80	11.8%	6.4%	2.9%	
	I often think about my friends and family dying	2.98	1.62	0.42	-0.76	23.6%	2.1%	3.2%	
View on a predestined lifespan		11.06	5.21	0.03	-0.85	13.5%	5.4%	3.2%	0.90
	I believe that lifespan is determined in advance	3.85	1.91	-0.04	-1.02	17.7%	10.3%	2.8%	
	I believe that lifespan is determined from the outset	3.65	1.90	0.07	-1.01	20.5%	9.0%	2.7%	
	I believe that life and death are determined by an invisible force (fate)	3.57	1.89	0.07	-1.03	22.6%	7.9%	2.7%	

DAI: Death Attitudes Inventory. SD: standard deviation. NA: not available.

The skewness and kurtosis were −0.46 to 0.62 and −1.05 to 0.37, respectively. This indicated that the DAI responses and subscales were normally distributed. The values of Cronbach’s α ranged from 0.83 (interest in death) to 0.93 (death anxiety/fear and death as a release). These values indicated good internal consistency and reliability of the DAI.

## Discussion

This is the first study to investigate and compare views on life and death among physicians, nurses, cancer patients, and the general population in Japan. We examined the psychometric properties of the DAI in a large population (n = 7,060) and found that the DAI was acceptable for use in this context, making the DAI the only validated instrument for use in measuring views on life and death in a large Japanese population.

Our first-order CFA analysis supported the seven-factor model proposed by Hirai et al., assuming that inter-correlation of the subscales gives a better model fit. Our second-order CFA model produced an acceptable fit; therefore, it is possible to to interpret the DAI in the context of TMT. The scarcity of studies on views on life and death in Japan makes interpretation of DAI findings difficult. Establishing a connection between the DAI and TMT would enable the utilization of the vast knowledge available from research on TMT. This kind of interconnectedness is promoted by advocates of TMT. Our multi-group analysis showed strong measurement invariance of the DAI across physicians, nurses, patients, and the general population. Therefore, the DAI could be successfully used in the context of medical decision-making.

Our regression analysis quantified the impact of age, sex, and social role on individual’s views on life and death. Previous studies showed that age, sex, culture, and occupation impact one’s views on life and death. Fortner et al. reported that elderly people has less fear of death than did younger people; fear of death was statistically similar between males and females [13]. Our results of the “death anxiety/fear” subscale were consistent with this. Powe et al. reported that cancer fatalism was most evident among older women [14]. Our results of the “view on a predestined lifespan” subscale seemed to be compatible with this finding.

Physicians and nurses scored lower on the "death anxiety/fear" and "death avoidance" subscales and higher on the “interest in death” subscale compared with the general population. This suggests that they employ an active coping style to perform their jobs providing care for patients, including those in the end-of-life stage. Seymour insisted that doctors had more difficulty than others in accepting dying, death, and bereavement as a natural part of the life cycle, with some regarding any death as a failure [15]. This may partly explain the lower scores on the “afterlife view” subscale in physicians.

Some subscale scores significantly differed between patients and the general population; however, the magnitude of difference was smaller than that of physicians and nurses. This could be explained in part by the fact that the majority of our patients were cancer survivors. It is also possible that formal knowledge of death and dying acquired through medical and nursing education have more of an impact on views on life and death than experience with a life-threatening disease such as cancer.

Finally, we investigated the relationship between religion and views on life and death. As expected, faith was correlated with 5 of the 7 DAI subscales. However, we did not detect a statistically significant correlation between faith and “death anxiety/fear” or “death avoidance.” The role of faith as a buffer for anxiety/fear of death needs to be investigated further.

### Limitations

There are several limitations to our study. First, our participants were not a random sample; they were all cancer patients attending the same radiology department at a university hospital. Further, most were at a point in their treatment where cure remained possible. The nurses were all staff at the same university hospital as our patients. This sampling limits the generalizability of our results. The general population included citizens of Tokyo. This sampling bias limits the external validity of our study. Second, the investigation period differed among the surveys. We cannot rule out the possibility of time-dependent confounding factors. Third, we did not investigate the patients' family members. Such data is expected to complement our understanding of views on life and death in terminal-care settings.

## Conclusions

This study is the first attempt to analyze the differences of views on life and death among physicians, nurses, cancer patients, and the general population in Japan. We revealed statistically significant differences in views on life and death by age, sex, and social role. Physicians and nurses scored higher on the “interest in death” subscale and scored lower on the "death anxiety/fear" and "death avoidance" subscales. While patients also scored higher on the “interest in death” subscale, their scores on the "death anxiety/fear" and "death avoidance" subscales had no significant difference. Higher interest in death shared among physicians, nurses and patients seems to encourage discussion about death among them. Discrepancy in the scores on "death anxiety/fear" and "death avoidance" subscales calls for medical practitioners' concern for patients. Influence of attitudes toward death upon coping with life threatening disease such as cancer warrants further investigation.

We provided further evidence for the DAI as a valid instrument for further investigations on views on life and death in Japan.

## Supporting information

S1 Dataset Dataset of this study. (XLSX)
